# Slow-Flow Venous Vascular Malformation in the Carotid Sheath

**DOI:** 10.7759/cureus.58638

**Published:** 2024-04-20

**Authors:** Pokhraj P Suthar, Mohamed Z Hussein, Sindhuja M K. Venkatraman, Miral Jhaveri, Santhosh Gaddikeri

**Affiliations:** 1 Department of Diagnostic Radiology and Nuclear Medicine, Rush University Medical Center, Chicago, USA; 2 Department of Pathology, Rush University Medical Center, Chicago, USA

**Keywords:** head & neck lesions, mri, ct, venous vascular malformation, carotid sheath, carotid space

## Abstract

Slow-flow venous vascular malformation is a benign lesion characterized by an abnormal but non-cancerous growth of capillaries. In the carotid space, a slow-flow venous vascular malformation is an exceptionally uncommon occurrence. Here, we present the case of a 58-year-old man who had been experiencing a slow-growing lump on the left side of his neck for the past four years. Upon conducting magnetic resonance imaging (MRI) of the neck soft tissues and computed tomography angiography (CTA) of the neck, imaging findings revealed a venous vascular malformation within the carotid sheath. Venous vascular malformation in the carotid space is rarely reported in the medical literature. The unique imaging findings for venous vascular malformation make our case distinct, which deviates from the more common entities usually encountered in this area, such as schwannoma or carotid body tumor. The atypical presentation of this case has brought about greater awareness among the medical community and readers alike.

## Introduction

Slow-flow venous vascular malformation is an exceptionally rare condition within the carotid space, and only a few cases have been documented in the medical literature. To diagnose this venous malformation in the carotid sheath accurately and avoid potential iatrogenic complications like intraoperative hemorrhage, imaging modalities such as magnetic resonance imaging (MRI) of the soft tissue neck with and without intravenous contrast and computed tomography angiography (CTA) of the neck play a crucial role [1]. These imaging techniques enable precise identification of the hemangioma, eliminating the need for unnecessary tissue sampling, which could otherwise pose risks. The differential diagnoses of carotid sheath hemangioma encompass various other conditions, including carotid body paraganglioma, vagal and sympathetic chain schwannomas, carotid sheath neurofibroma, carotid artery aneurysm, and carotid space lymphoma. In this article, we present an interesting case of a 58-year-old man with a history of a slow-growing left-side neck lump with an MRI of the soft tissue neck and CTA of the neck findings of carotid sheath venous vascular malformation.

## Case presentation

A 58-year-old man presented with a four-year history of a slow-growing lump on the left side of his neck. During examination, the patient denied any history of hoarseness of voice, dysphagia, shortness of breath, palpitation, or chest pain. Vital signs were all within normal ranges, including blood pressure (120/75 mmHg), temperature (97.4°F or 36.3°C), heart rate (69 beats per minute), respiratory rate (17 per minute), and SpO_2_ at 99%. A soft tissue non-pulsatile mass was palpable on the left side of the neck, at the level of the hyoid bone. The oropharynx appeared clear without any signs of swelling, exudate, or erythema. The rest of the physical examination revealed no abnormalities. The patient's prior psychiatric, surgical, and family histories were unremarkable, and there was no recent history of travel, camping, hiking, or vaccinations. Laboratory workup showed an elevated point-of-care serum glucose level of 116 mg/dL (normal reference range: 60-99 mg/dL) and a marginally elevated hemoglobin A1C level of 5.7% (normal reference range: 0.0%-5.6%). Otherwise, the rest of the routine laboratory work was within normal limits.

**Figure 1:**
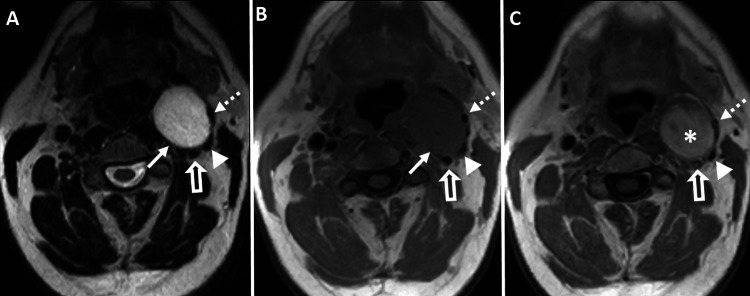
MRI of the soft tissue neck Axial images: (A) T2-weighted (repetition time msec/echo time msec, 11636/126, 5-mm section thickness), (B) unenhanced T1-weighted (repetition time msec/echo time msec, 634/16, 5-mm section thickness), and (C) contrast-enhanced T1-weighted (repetition time msec/echo time msec, 634/16, 5-mm section thickness) after intravenous injection of 16 mL of gadoteridol (ProHance; Bracco). The MRI images demonstrate a well-circumscribed, oval-shaped, homogeneously T2 hyperintense, and T1 hypointense lesion in the left carotid space (solid white arrows in images A and B). The mass causes left postero-lateral displacement of the left internal carotid artery (open white arrows in images A-C), left external carotid artery (dashed white arrowheads in images A-C), and internal jugular vein (solid white arrowheads in images A-C). The lesion exhibits near-complete contrast enhancement (white asterisks in image C) with minimal splaying of the internal and external carotid arteries.

MRI of the soft tissue neck with and without intravenous contrast was performed to assess the lesion. The imaging revealed a well-defined unilocular oval-shaped mass, measuring 4 cm (anteroposterior) x 3.3 cm (transverse) x 4.1 cm (craniocaudal), situated in the left carotid space. The lesion appeared homogeneously T1 isointense and T2 hyperintense. It caused a posterolateral displacement of the left internal carotid and internal jugular vein (IJV) as well as a ventrolateral displacement of the external carotid artery and its branches. The mass exhibited avid contrast enhancement, with minimal splaying of the internal and external carotid arteries. Notably, there was no intrinsic T1 hyperintensity, T2 heterogeneity, or evidence of local invasion (Figure 1). Based on the MRI findings, the initial diagnosis of sympathetic chain schwannoma was considered. Other potential differential diagnoses included carotid body paraganglioma, vagal schwannoma, or lymphoma.

**Figure 2:**
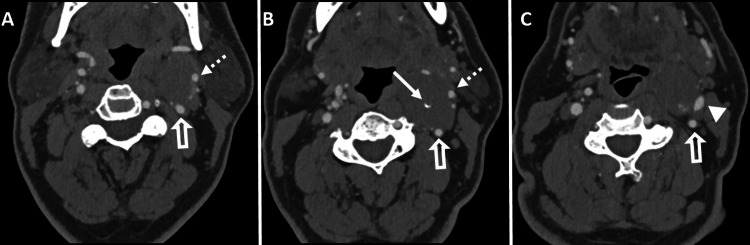
CTA of the neck Axial images were taken after the intravenous injection of 80 mL of iopamidol (Isovue-370; Bracco). The CTA reveals a soft tissue density lesion in the left carotid space with a few punctate calcifications/phleboliths (solid white arrow in B) and mild peripheral enhancement in the arterial phase. There is posterolateral displacement of the left external carotid artery (indicated by dashed arrows in A and B), left internal carotid artery (open white arrows in A-C), and internal jugular vein (solid white arrowhead in C). CTA: Computed tomography angiography.

**Figure 3:**
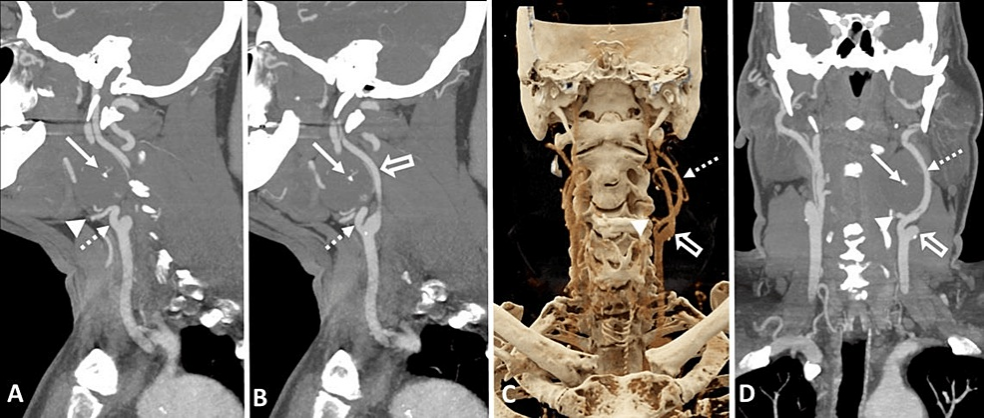
CTA of the neck after intravenous injection of 80 mL of Iopamidol (Isovue-370; Bracco) (A and B) Sagittal maximum intensity projection (MIP) images. (C) Cinematic volume rendering from CTA (Siemens Healthineers). (D) Coronal MIP images. The CTA illustrates a soft tissue density lesion in the left carotid space with a few punctate calcifications/phleboliths (solid white arrows in A, B, and D) and mild peripheral enhancement during the arterial phase. The left internal carotid artery is posterolaterally displaced (open white arrows in B-D). The ascending pharyngeal branch (solid white arrowhead in A and C) of the left external carotid artery (dashed white arrows in A-D) courses along the inferior aspect of the lesion. CTA: Computed tomography angiography.

**Figure 4:**
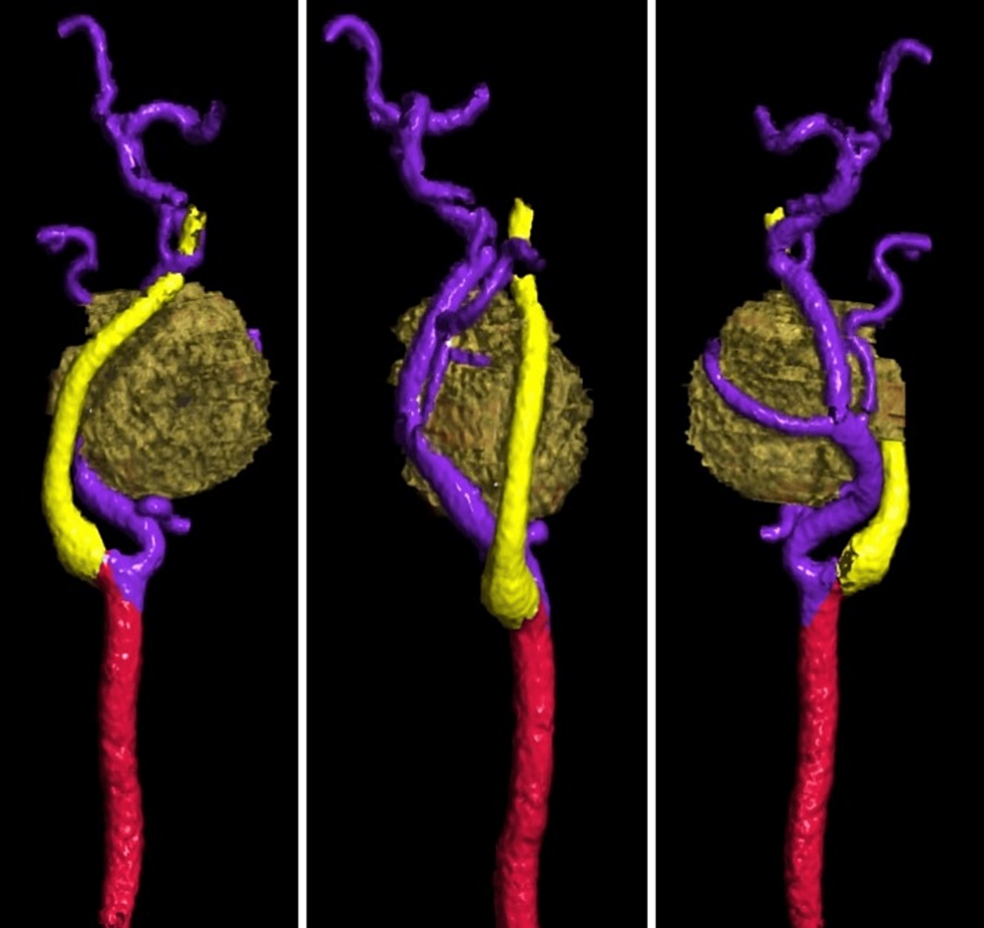
3D color-coded image of left carotids from CTA of the neck (Siemens Healthineers) A soft tissue density lesion was observed in the left carotid space with its relation to the left internal carotid artery (yellow color-coded), left external carotid artery (purple color-coded), and left common carotid artery (red color-coded). CTA: Computed tomography angiography.

**Figure 5:**
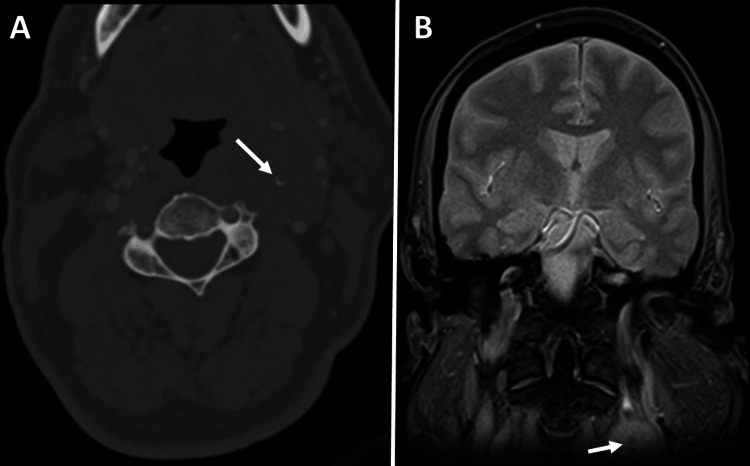
(A) Axial CTA of the neck in bone window setting demonstrates central punctate calcification (white arrow in A). (B) The coronal GRE image of the brain demonstrates partially included low signal intensity focus (white arrow in B) in the partially imaged lesion of left carotid space, which corresponds to the calcification seen in the CT scan. CTA: Computed tomography angiography; GRE: Gradient echo sequences.

To further evaluate the lesion and plan for elective surgical excision, the patient underwent pre-surgical CTA of the neck. The CTA demonstrated a well-circumscribed hypodense soft tissue mass in the left carotid space, exhibiting mild peripheral pooling of contrast in the arterial phase. The left external carotid artery (ECA) and its branches were displaced ventrally and laterally by the mass. Additionally, the ascending pharyngeal branch coursed along the inferior and lateral aspects of the lesion. Moreover, the mass caused the posterolateral displacement of the left internal carotid artery (ICA) and IJV, while the bilateral carotid and vertebral arteries remained patent (Figures 2-4). Additionally, few punctate calcifications were found within the lesions (Figure 5).

The patient was administered general orotracheal anesthesia, and a left upper neck incision was performed under sterile conditions to access the left carotid space. During the procedure, a red-purple spherical discolored mass at the carotid bifurcation was observed. Intraoperatively, it was found that the mass was adherent to the ECA, which led to the decision to sacrifice the ECA above the lingual artery. Notably, there was no apparent connection between the mass and any nerve origin. The mass was resected with special attention to ensuring the safety of the ICA.

**Figure 6:**
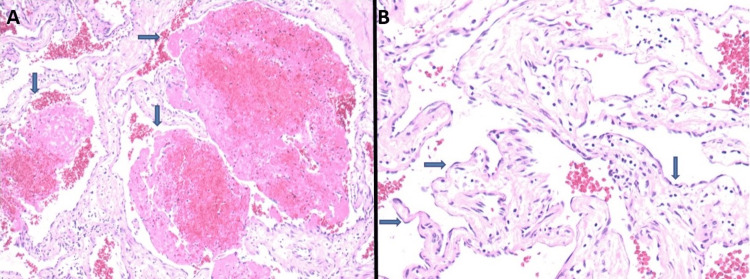
Photomicrograph of a surgical specimen (A) The photomicrograph depicts dilated and congested blood vessels (blue arrows) (hematoxylin-eosin stain; magnification ×4). (B) These blood vessels are lined by a single layer of flat, bland-appearing endothelial cells (blue arrows) without atypia or significant mitotic activity (hematoxylin-eosin stain; magnification ×20).

On histopathological examination, the findings revealed dilated and congested, thin-walled blood vessels lined by a single layer of flat, bland-appearing endothelial cells. These cells showed no signs of atypia or significant mitotic activity, which is consistent with a diagnosis of slow-flow vascular malformation of the carotid sheath (Figure 6).

## Discussion

The ISSVA classification, developed by the International Society for the Study of Vascular Anomalies, serves as a globally recognized framework for categorizing all vascular malformations and tumors, employing consistent nomenclature [2]. Originating from the 1982 classification by Mulliken and Glowacki, it has undergone updates to incorporate the identification of causal genetic mutations. The latest revision in May 2018 refined the classification [2]. Among slow-flow lesions, venous malformations predominate, typically manifesting in mid to late childhood. These lesions are characterized by their continuous, soft, and compressible nature, involving multiple layers. They often align with muscle groups, follow nerve pathways, or course along major vessels. Notably, venous malformations exhibit responsiveness to changes in venous flow, such as during Valsalva maneuvers, or in some cases, compression of the ipsilateral jugular vein, particularly when located in the head and neck region [2]. In the literature, reports of cavernous hemangiomas/slow-flow vascular malformation of the carotid sheath are rare. Our search on PubMed revealed the existence of only five documented cases, underscoring the rarity of this entity [3-7]. These lesions may involve deeper structures, and unlike capillary hemangiomas, they frequently do not regress, often requiring surgical treatment [4]. Histologically, these lesions are characterized by large, cavernous vascular spaces separated by scant connective tissue stroma [8].

The MRI reveals a soft tissue mass in the neck area, occasionally exhibiting flow voids indicative of feeding vessels. In T1-weighted images, the lesion appears isointense or hypointense to muscle, while in T2-weighted images, it appears hyperintense. The enhancement pattern is characterized by peripheral nodular avid enhancement in the early phase, followed by centripetal filling on delayed images. On CTA, the lesion demonstrates avid peripheral enhancement during the arterial phase, with gradual centripetal pooling of the contrast in the venous phase. The presence of a phlebolith can aid in distinguishing this condition from other vascular masses in the carotid sheath. During conventional catheter angiography, the mass exhibits persistent, dense contrast staining. The enhancement pattern is similar to that observed in cavernous orbital hemangioma/slow-flow vascular malformation [1].

The differential diagnoses of carotid sheath slow-flow vascular malformation include carotid body tumor/paraganglioma, vagal or sympathetic chain schwannomas, carotid sheath neurofibroma, carotid artery aneurysms, carotid space lymphoma, carotid space infection, and carotid sheath meningioma (Table 1). Paragangliomas typically originate within the paraganglia of the head and neck, often associated with the branches of the carotid body, glossopharyngeal, and vagus nerves. Carotid body tumors are located at the carotid bifurcation and exhibit characteristic splaying of the ICA and ECA. In contrast to carotid sheath hemangioma/slow-flow venous vascular malformation, they demonstrate intense early enhancement following contrast administration, with features of arteriovenous shunting. Like hemangiomas, they appear T2 hyperintense and may occasionally exhibit a "salt and pepper" appearance on T1-weighted images, indicating a combination of punctate subacute hemorrhage (salt) and flow voids (pepper) [4].

**Table 1:** Differential diagnosis of carotid space lesions MRI: Magnetic resonance imaging; CT: Computed tomography; ECA: External carotid artery; ICA: Internal carotid artery; IJV: Internal jugular vein; MRA: Magnetic resonance angiography.

Differentials	Imaging features
Carotid body tumor/paraganglioma	Located at the carotid bifurcation and exhibits characteristic splaying of ICA and ECA. They demonstrate intense early enhancement following contrast administration, with features of arteriovenous shunting. Like hemangiomas/slow-flow venous vascular malformation, they appear T2 hyperintense and may occasionally exhibit a "salt-and-pepper appearance" on T1-weighted images, indicating a combination of punctate subacute hemorrhage (salt) and flow voids (pepper).
Vagal schwannomas	Typically displace the ICA anteriorly and the internal jugular vein posteriorly. Intense heterogeneous enhancement and heterogeneously hyperintense on T2-weighted image, depending on the concentration of Antoni B cells and/or cystic degeneration.
Sympathetic chain schwannomas	Displace the ICA and IJV in an anterior and lateral direction. The separation of the IJV and ICA often aids in distinguishing vagal and sympathetic chain schwannomas.
Carotid sheath neurofibroma	Carotid sheath neurofibromas can be differentiated from schwannomas by their tapered end, which resembles the parent nerve "entering and exiting." A characteristic target sign or fish-eye appearance, with a high T2 signal peripherally and a low central T2 signal, favors the diagnosis of neurofibroma.
Carotid artery aneurysms	Contrast-enhanced CT reveals calcification of the peripheral wall, giving it an "eggshell" appearance, and exhibits arterial enhancement. Flow-time-of-flight MRA shows flow-related enhancement within the aneurysmal sac.
Carotid space lymphoma	It encases the carotid arteries, unlike slow-flow venous vascular malformation, which displaces them. On MRI, it appears homogeneously T1 hypointense and T2 intermediate intensity. The mass uniformly enhances and demonstrates restricted diffusion.
Carotid sheath infection	Head and neck infections can cause cervical adenitis, typically resulting from viral upper respiratory infections but with various potential causes. Enlarged lymph nodes, exceeding 1 cm, may progress to suppurative lymph nodes with liquefactive necrosis. If untreated, these nodes may rupture into deep neck spaces. Common causative agents include *Staphylococcus aureus* and group A streptococcus. For quick evaluation, especially in emergencies, contrast-enhanced CT scans are preferred.
Carotid sheath meningioma	Meningioma is rare in the carotid sheath. Meningioma demonstrates calcification and contrast enhancement. Encasement of major carotid vessels and cranial nerves gives the "tram-track appearance."

Vagal nerve schwannomas typically separate the ICA anteriorly and the IJV posteriorly. Unlike slow-flow vascular malformation, vagal schwannomas demonstrate intense heterogeneous enhancement. Their appearance on T2-weighted images is heterogeneously hyperintense, depending on the concentration of Antoni B cells and/or cystic degeneration. Sympathetic chain schwannomas, on the other hand, tend to displace the ICA and IJV in an anterior and lateral direction. The separation of the IJV and ICA often aids in distinguishing vagal and sympathetic chain schwannomas [9]. Carotid sheath neurofibromas can be differentiated from schwannomas by their tapered end, which resembles the parent nerve "entering and exiting." A characteristic target sign or fish-eye appearance, with a high T2 signal peripherally and a low central T2 signal, favors the diagnosis of neurofibroma [9].

Carotid space lymphoma is a rare condition that encases the carotid arteries, unlike slow-flow venous vascular malformation, which displaces them. On MRI, it appears homogeneously hypointense on T1-weighted and intermediate in intensity on T2-weighted sequences. The mass uniformly enhances and demonstrates restricted diffusion [10]. Carotid artery aneurysms are infrequent lesions and are typically asymptomatic, although neurologic manifestations have been reported due to emboli. Contrast-enhanced CT reveals calcification of the peripheral wall, giving it an "eggshell" appearance, and exhibits arterial enhancement. Flow-time-of-flight MRA shows flow-related enhancement within the aneurysmal sac.

The treatment for slow-flow vascular malformation of the carotid sheath is surgical removal [7]. Preoperatively, angiography and embolization may be used to expedite involution and reduce size. Other treatment options, such as steroids, chemotherapy, radiotherapy, sclerotherapy with foam injection, and embolization, have been described, but their efficacy remains uncertain [5].

## Conclusions

Carotid sheath slow-flow venous malformation is considered one of the differentials for carotid space lesions, although rare. As per the recently revised ISSVA classification, vascular anomalies can be categorized into vascular tumors and vascular malformations. The former refers to authentic proliferative neoplasms, while the latter denotes defects in vascular morphogenesis. MRI of the soft tissue neck and CTA of the neck are non-invasive imaging modalities, which help diagnose and avoid unnecessary tissue sampling, preventing hazardous iatrogenic complications. The differential diagnoses of carotid sheath hemangioma include carotid body paraganglioma, vagal and sympathetic chain schwannomas, carotid sheath neurofibroma, carotid artery aneurysm, and carotid space lymphoma. Our case is unique with classic imaging findings, which are not routinely encountered in day-to-day practice. As there are only a few documented cases in the literature to date, this report aims to increase awareness of this rare entity among the readers.
